# Is a Negative Attentional Bias in Individuals with Autism Spectrum Disorder Explained by Comorbid Depression? An Eye-Tracking Study

**DOI:** 10.1007/s10803-021-04880-6

**Published:** 2021-01-24

**Authors:** M. Annemiek Bergman, Janna N. Vrijsen, Mike Rinck, Iris van Oostrom, Cornelis C. Kan, Rose M. Collard, Philip van Eijndhoven, Constance Th. W. M. Vissers, Aart H. Schene

**Affiliations:** 1 Department of Psychiatry, Radboud University Medical Centre, Radboudumc, 9101, 6500 HB Nijmegen, The Netherlands; 2 Donders Institute for Brain, Cognition and Behavior, Nijmegen, The Netherlands; 3 Depression Expertise Center, Pro Persona Mental Health Care, Nijmegen, The Netherlands; 4 Radboud University Nijmegen, Behavioural Science Institute, Nijmegen, The Netherlands; 5 NeuroCare, Nijmegen, The Netherlands; 6 Royal Dutch Kentalis, Kentalis Academy, Sint-Michielsgestel, The Netherlands

**Keywords:** Autism spectrum disorder, Depression, Attentional bias, Comorbidity, Cognitive bias

## Abstract

Heightened attention towards negative information is characteristic of depression. Evidence is emerging for a negative attentional bias in Autism spectrum disorder (ASD), perhaps driven by the high comorbidity between ASD and depression. We investigated whether ASD is characterised by a negative attentional bias and whether this can be explained by comorbid (sub) clinical depression. Participants (*n* = 116) with current (CD) or remitted depression (RD) and/or ASD, and 64 controls viewed positively and negatively valenced (non-)social pictures. Groups were compared on three components of visual attention using linear mixed models. Both CD individuals with and without ASD, but not remitted depressed and never-depressed ASD individuals showed a negative bias, suggesting that negative attentional bias might be a depressive state-specific marker for depression in ASD.

Autism spectrum disorder (ASD) is a neurodevelopmental disorder characterised by a triad of deficits involving communication, reciprocal social interactions, and restricted and repetitive behaviours and interests (American Psychiatric Association 2013). The estimated prevalence of ASD is almost 1% in the general population (Baxter et al. 2015). ASD frequently co-occurs with other mental disorders such as attention-deficit/hyperactivity disorder (ADHD), anxiety disorders and depression (Matson and Nebel-Schwalm 2007; Simonoff et al. 2008). For instance, the co-occurence of depression in individuals with ASD is four times as high as in neurotypical individuals (Hudson et al. 2019) and ASD individuals, moreover, tend to experience high levels of depressive symptoms even when a clinical diagnosis of depression is absent (Gotham et al. 2015). This comorbidity is associated with functional burden and clinical implication (Joshi et al. 2013; Mazefsky et al. 2012) and higher suicidal risk (De-la-Iglesia and Olivar 2015).

Depression in ASD is often underrecognised and, thus, undertreated (Chandrasekhar and Sikich 2015). Characteristics of ASD can complicate the assessment and diagnosis of depressive symptoms in ASD, such as concentration problems and difficulties in communicating affect through facial expressions or intonation (Stewart et al. 2006). Despite the high prevalence and impact of depression in ASD, the factors contributing to the comorbidity between these disorders are not well understood. This lack of knowledge hinders innovations in the diagnostics and treatment of comorbid ASD and depression. In this study, we aim to expand this knowledge by investigating negative attentional bias, a well-known cognitive vulnerability factor for developing and maintaining depression (De Raedt and Koster 2010; Gotlib and Joormann 2010), in individuals with ASD who either have or have not developed a comorbid depression.

According to a prominent cognitive model of depression (Beck 2008; Beck and Bredemeier 2016), the experience of adverse events during childhood may contribute to the development of dysfunctional assumptions about oneself, the future, and the world. These assumptions are integrated into cognitive schemata. When activated (e.g., by stress), these schemata affect how information is processed, which may result in cognitive biases (Beck 2008). These biases are studied in different cognitive domains, such as attention, interpretation, and memory (LeMoult and Gotlib 2018; Mathews and MacLeod 2005).

Negatively biased attentional processing is the automatic tendency to focus more on negative information and is a hallmark feature of depression (Beck 1967; Beck and Bredemeier 2016; Bower 1981; LeMoult and Gotlib 2019; Williams et al. 1988). This feature has generally been examined in depression using behavioural tasks such as the dot-probe task (e.g., Peckham et al. 2010), the emotional Stroop task (e.g., Peckham et al. 2010), the exogenous cuing paradigm (e.g., Koster et al. 2005), or the visual search task (e.g., Rinck and Becker 2005). However, a main methodological limitation of these tasks is their reliance on reaction times; these are susceptible to confounding influences of manual reactions (Mathews et al. 1996). An eye-tracker can be used to measure attentional bias more continuously and directly (Armstrong and Olatunji 2012), and is therefore a good alternative to the reaction time tasks (Waechter et al. 2014). Moreover, using an eye-tracker enables researchers to examine attention at several stages of processing, from initial engagement and shifting of attention to overall engagement (Armstrong and Olatunji 2012). An eye-tracking task was therefore employed in the current study.

Depressed individuals show increased maintained attention (i.e., overall engagement) towards negative and away from positive information compared to never-depressed individuals, which is interpreted as a difficulty in disengaging from negative information (e.g., Armstrong and Olatunji 2012; Gotlib and Joormann 2010; Kellough et al. 2008; Peckham et al. 2010). Discrepancies exist in the attentional bias literature for individuals who have recovered from a depressive episode (i.e., remitted depression). A number of studies have shown similar attentional bias patterns using reaction time tasks in both current and remitted depressed individuals; namely, either more negative or less positive attentional bias (e.g., Peckham et al. 2010; Joormann and Gotlib 2007). Additionally, Sears et al. (2011) found in their eye-tracking study negative bias in the first stage but not in later stages of processing (i.e., measured by the number of fixations) in remitted depressed and current dysphoric individuals compared to never-depressed individuals. In contrast, other eye-tracking studies found no difference in initial attention (i.e., first fixation location) between remitted and never-depressed individuals (e.g., Isaac et al. 2014; Li et al. 2016). However, Isaac et al. (2014) found evidence for a positive bias on overall gaze duration in both remitted and never-depressed individuals, as opposed to Li et al. (2016) demonstrating a less positive attentional bias for the remitted depressed individuals, but no differences in negative bias compared with the healthy controls. Given that the evidence for negative attentional bias in remitted depressed patients is mixed and findings may differ depending on the eye-tracking index used (e.g., overall gaze duration, number of fixation), we aimed to differentiate between currently and remitted depressed patients on various frequently used eye-tracking indices.

Children with ASD are more prone to encounter adverse events compared to neurotypical children, such as being frequently victimised by peers (Zablotsky et al. 2014) as well as having other types of social difficulties such as loneliness (Bauminger et al. 2003). As described in the cognitive model by Beck (2008), adverse events during childhood can result in negative cognitive biases. The frequent experience of adverse childhood events may form the basis for the development of negative attentional bias in ASD. Research on cognitive biases in ASD is limited (Bergman et al. 2020), with the available studies mainly showing reduced attentional bias towards social information (e.g., persons, faces, eyes) relative to non-depressed individuals (Chita-Tegmark 2016; Dubey et al. 2017; Sasson et al. 2008), although contradictory findings have also been reported (for a review, see Guillon et al. 2014). This makes sense because ASD is characterised by impairments in reciprocal social interaction (Dawson et al. 2004). When it comes to affective stimuli, the few studies available show equivocal results, with most evidence (mainly from reaction time tasks) in favour of a more positive bias in ASD compared to individuals without ASD (see review by Bergman et al. 2020). A more recent eye-tracking study, however, found that both individuals with ASD and depressed individuals oriented faster to negative stimuli and spent less time overall on positive stimuli than non-clinical individuals (Unruh et al. 2018).

In the current study, we aimed to examine if individuals with ASD show a negative attentional bias and whether the negative bias is explained by comorbid clinical and subclinical depression. For this purpose, we employed a free-viewing eye-tracking task assessing attentional bias for positive and negative, social and non-social stimuli. Five groups of participants were compared using the eye-tracking indices: Individuals diagnosed with ASD (ASD), currently depressed individuals with no ASD (CD), remitted depressed individuals with no ASD (RD), individuals with ASD and comorbid current and/or remitted depression (ASD+CD/RD), and healthy controls (HC). We expected that CD would attend longer to negative stimuli compared to positive stimuli, whereas HC were expected to show a positive attentional bias. RD individuals were expected to show a negative bias, albeit somewhat weaker than CD (cf. Isaac et al. 2014; Peckham et al. 2010). Based on previous eye-tracking studies (Chita-Tegmark 2016; Unruh et al. 2018), participants with ASD—both with and without concurrent depression—were expected to exhibit a negative attentional bias, specifically for non-social stimuli. By comparing participants with ASD and RD to those with ASD and CD, we examined the trait-like feature of attentional bias in ASD. Since attentional bias for valenced information in ASD and ASD with comorbid depression has not been frequently investigated, we additionally examined if a similar pattern of attentional bias is present in attentional indices besides overall engagement (i.e., initial engagement and shifting). This will provide a more complete and sensitive overview of the possible presence of attentional bias at different stages of information processing of valenced information in ASD (and depression).

## Methods

### Participants

This study is part of the MIND-Set study (Measuring Integrated Novel Dimensions in Neurodevelopmental and Stress-related Mental Disorders): An ongoing observational cross-sectional study that takes place at the outpatient unit of the Psychiatry department of Radboud university medical center (Radboudumc), Nijmegen, the Netherlands. The study has been approved by the Ethical Review Board of the Radboudumc. Adult clinical patients (18 and older) with a clinical diagnosis of a stress-related disorder (mood disorder, anxiety disorder, and/or substance use disorders [SUD]) and/or a neurodevelopmental disorder (ASD and/or ADHD) were eligible to participate.

An additional healthy control (HC) group was recruited by advertising in the community (e.g., social media and websites), via the Radboud Research Participation System as well as verbally through researchers’ personal networks. In this group, the absence of a lifetime history of the aforementioned mental disorders was verified via a telephone screening interview, using the same measurement instruments for the MIND-Set population as described below in the Clinical and Demographic Characteristics section. All participants had normal or corrected to-normal vision. Participants with a current psychosis, sensorimotor handicaps, an estimate IQ below 70, insufficient mastery of the Dutch language, epilepsy (only for the eye-tracker task) or participants who were mentally incompetent to sign informed consent were excluded. All participants signed informed consent before taking part.

A subset of the MIND-Set sample used in the current study which was collected from August 2016 to May 2018 and consisted of the following five final groups: Participants diagnosed with ASD (ASD; *n* = 15), currently depressed participants with no ASD (CD; *n* = 40), remitted depressed participants with no ASD (RD; *n* = 24), participants with ASD and comorbid current and/or remitted depression (ASD+CD/RD; *n* = 37), and healthy control participants (HC; *n* = 64). Eye-tracking data from nine participants from the ASD+CD/RD group, nine from the CD, and one participant from the HC group were discarded due to excessive artifacts and calibration problems*.* A patient was given the diagnosis of remitted depression if at least one previous depressive episode was present and the patient was currently not meeting the Diagnostic and Statistical Manual of Mental Disorders, fourth edition (DSM-IV) criteria for a major depressive disorder. Remitted depression included full and partial remission (one till four depressive symptoms according to the DSM-IV). Previous episodes were assessed with the Structured Clinical Interview for DSM-IV Axis I Disorders (SCID-I; First et al. 1996). Subsequently, to examine whether the bias in ASD remains after remission, the ASD+CD and ASD+RD groups were compared in additional post hoc analyses.

To increase the validity and generalizability of our results, we used a naturalistic clinical patient sample as included in the larger MIND-Set population. Thus, the participants in the subsample of this study could, besides (remitted) depression and/or ASD, have additional comorbid mental disorder(s), such as ADHD, anxiety disorder(s) and/or SUD. Participants with only a diagnosis of dysthymia or bipolar disorder were excluded from this subsample. A final subsample of 116 participants with ASD and/or depression and 64 HC was included in the analyses. All participants were between 18 and 65 years of age. For the demographic variables of the final groups, see Table 1; for the comorbid disorders present in the participants with ASD and/or depression, see Table 2.

**Table 1:** Group comparisons on demographic variables (means and standard deviations [SD]), including test statistics for the group comparisons

Group						
Variable	ASD (*n* = 15)	CD (*n* = 40)	RD (*n* = 24)	ASD + CD/RD (*n* = 37)	HC (*n* = 64)	Group comparisons
Gender, female (%)	47	45	42	37	58	χ^2^ (4) = 4.54, *p* = 0.338
Age, mean (SD)	39 (12.06)	43 (14.09)	41 (10.79)	37 (13.46)	35 (14.69)	*F *(4, 175) = 2.33, *p* = 0.058
Education level^a^						χ^2^ (8) = 17.67, *p* = 0.024
Low (%)	13	28	21	15	2	
Middle (%)	40	30	29	41	34	
High (%)	40	35	50	35	58	
IDS-SR	25 (13.84)	43 (11.01)	27 (10.61)	32 (11.13)	5 (4.34)	*F *(4, 171) = 105. 25, *p* < 0.001

*ASD* autism spectrum disorder, *CD* current depression, *RD* remitted depression, *ASD* +*CD/RD* autism spectrum disorder with current and/or remitted depression, *HC* healthy controls, *IDS-SR* inventory of depressive symptomatology-self rated

^a^Adjusted classification based on the classification by Ikram et al. (2014). Low: no education or elementary education and lower vocational and general secondary education combined. Middle: intermediate vocational and higher secondary education. High: higher vocational education or university

**Table 2:** Prevalence of comorbid mental disorders in the ASD and/or depression participants

Group				
Comorbid disorder	ASD (*n* = 15)	CD (*n* = 40)	RD (*n* = 24)	ASD+CD/RD (*n* = 37)
ADHD (%)	27	20	50	27
Anxiety disorder (%)	20	25	38	38
SUD (%)	0	23	17	0
Number of diagnoses, indicating level of comorbidity (%)	1 = 53; 2 = 27, 3 ≥ 13	1 = 50, 2 = 28, 3 ≥ 23	1 = 13, 2 = 54, 3 ≥ 33	2 = 43, 3 ≥ 57

*ADHD* attention-deficit/hyperactivity disorder, *SUD* substance use disorder

### Clinical and Demographic Characteristics

The MIND-Set study was conducted during the transition period from the DSM-IV to DSM-5. All individuals were diagnosed and classified by a trained and experienced clinician. Stress-related disorders and ADHD were classified according to DSM-IV and ASD according to DSM-5. Mood disorders and anxiety disorders were assessed with the SCID-I (First et al. 1996) and SUD with the Measurements in the Addictions for Triage and Evaluation and criminality (MATE-Crimi; Schippers et al. 2010). The SCID-I was also used to exclude individuals with psychotic disorders. Neurodevelopmental disorders were assessed during a two-step diagnostic screening procedure, using the World Health Organization Adult ADHD Self-report Scale (ASRS)-short version for ADHD (Kim et al. 2013) and the Autism-Spectrum Quotient-50 (AQ-50; Baron-Cohen et al. 2001) for ASD screening. When screening was positive (six items, cut-off  > 3), we used the Diagnostic Interview for ADHD in Adults Version 2.0 (DIVA 2.0; Kooij and Francken 2010) for ADHD diagnosis. When an individual scored positive on the AQ-50 (50 items, cut-off  > 25), we used the Dutch Interview for ASD in Adults (in Dutch: Nederlands Interview ten behoeve van Diagnostiek Autismespectrumstoornissen bij volwassenen; NIDA; Vuijk 2016) for ASD diagnosis. If possible, both the DIVA and NIDA were completed in the presence of a partner and/or family member of the individual to be able to retrospectively gather information on a broad range of symptoms in childhood and adulthood, following the Dutch guidelines (Kan 2013). If an individual was previously diagnosed with ASD or (an) other disorder(s) by another institution, the diagnostic information was retrieved and examined by the treating clinician (this was applicable for 19% of the partcipants with ASD included in this study). In addition, every participant was asked to complete the Inventory of Depressive Symptomatology (IDS-SR; Rush et al. 1996) to measure depressive symptom levels. Sociodemographic information concerning the participant’s gender, age, and education level was acquired by using online questionnaires.

### Materials and Apparatus

#### Free-Viewing Task

The free viewing task comprised of 96 pictures selected from the International Affective Picture System (IAPS; Lang et al. 1997) and the Nencki Affective Picture System (NAPS; Marchewka et al. 2014). Pictures were selected to fit a social stimulus category (i.e., one or more individuals) and a non-social stimulus category (i.e., landscapes, animals and objects). Moreover, based on the valence ratings, pictures in the negative (*M* = 2.51, SD = 0.42) and positive (*M* = 7.79, SD = 0.33) categories were selected. For both the social and non-social pictures, half of all the stimuli were positively valenced and the other half were negatively valenced. These stimulus categories were chosen to capture both attentional bias for negative information and bias for social information in ASD, as previously demonstrated (Chita-Tegmark 2016; LeMoult and Gotlib 2018). The final stimulus groups were thus: negative non-social (NNS), negative social (NS), positive non-social (PNS), and positive social (PS). An independent-samples t-test was performed on valence ratings of negative (NNS & NS) and positive (PNS & PS) pictures, demonstrating a significant effect of valence (*t *(95) = 19.06, *p* < 0.001). A separate ANOVA conducted for arousal ratings of NNS (*M* = 6.78, SD = 0.42), NS (*M* = 6.36, SD = 0.81), PNS (*M* = 3.79, SD = 0.82), and PS (*M* = 4.73, SD = 0.72) showed a significant effect of arousal (*F* (3, 92) = 92.79, *p* < 0.001). Post hoc analyses demonstrated that all pair-wise comparisons were significant (*p* < 0.048). Negative stimuli, in general, were rated with higher arousal levels compared to positive stimuli, since, for example, a picture of a tropical island is less arousing than a mutilated animal. In addition, high arousing (i.e., threatening) stimuli are mainly associated with biased attentional processing in anxiety disorders and not in depression (Peckham et al. 2010). Since we are only interested in attentional bias for valence, we conducted the experiment with the set of stimuli as included in this study.

**Fig. 1:**
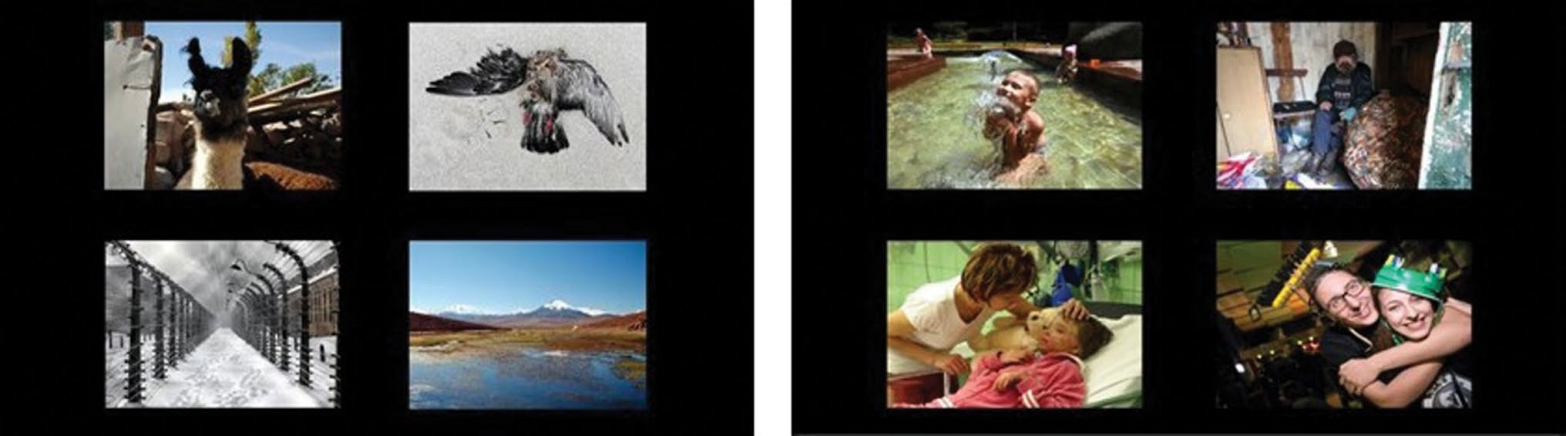
Left: an example slide from the non-social block, in which two positively valenced pictures (top left and bottom right) and two negatively valenced pictures are depicted (top right and bottom left). Right: an example slide from the social block, in which two positively valenced pictures (top left and bottom right) and two negatively valenced pictures (top right and bottom left) are depicted

Each trial began with a 1000 ms centrally presented fixation cross, followed by the simultaneous presentation of one slide (i.e., one trial) containing four pictures for 30 s. A total of 12 trials per block (i.e., a block contained all the social or non-social stimuli) were presented. Two pictures of the same valence were not presented consecutively. Blocks were presented in a random order across participants and not per participant group. For an example of a slide, see Fig. 1.

#### Eye-Tracker

A remote eye-tracking system (SMI RED500) was used to measure participants’ eye movements; a free range of head movements was allowed. The sampling rate was 500 Hz. Data were collected using a velocity-based algorithm with a minimum fixation duration threshold of 100 ms and a peak velocity threshold of 40◦/s. The areas of interest (AOIs) were identified for each trial, and corresponded to the total area for each of the four pictures plus the areas in the outer corners of the four pictures to take recording noises into account. A total of four AOIs were constructed, corresponding to the picture categories: positive social, negative social, positive non-social, and negative non-social. Eye-tracking data were preprocessed using SMI BeGaze Version 3.7 (SensoMotoric Instruments, Inc., Teltow, Germany). The data were visually inspected for abnormalities and checked for distributional anomalies; these were not found in the current sample.

### Procedure

Prior to the start of the eye-tracking task, participants were placed in a height-adjustable chair approximately 60 cm in front of the table-mounted eye-tracker with a 22″ Dell TFT-monitor on which the stimuli were presented. The experiment started once the nine-point calibration procedure was completed successfully (i.e., the mean of the error was 1.5◦ or less of the visual angle for each calibration point; in line with García-Blanco et al. 2014). Between blocks, the calibration procedure was repeated. The participants were instructed to focus their gaze on the fixation cross. Upon presentation of the four pictures, participants were instructed to view the pictures naturally in preparation of a recognition task. The eye-tracking task consisted of two parts: a free-viewing task and a recognition task. Beforehand, the participants were told that the recognition task would be administered after the free-viewing task. The recognition task contained the same stimuli and had to be completed by the participant to obscure the nature of the task. In this task, participants were instructed to click on the image that had changed from its initial location in the free-viewing task. In the current study, we present the results of only the free-viewing task. The total duration of the eye-tracking task including the calibration procedures was approximately 20 min.

### Eye Movement Data Preparation

A distinction can be made between different components of attentional bias (Cisler and Koster 2010; Yiend 2010). Based on prior studies (e.g., García-Blanco et al. 2014; Isaac et al. 2014; Mo et al. 2019), the following three attentional indices were computed: (1) *Overall engagement:* total gaze duration (ms) per AOI (i.e., the total duration in ms that each participant’s gaze remained fixated within the boundaries of a given AOI). (2) *Initial engagement*: the location of the first fixation on a given AOI in each trial. (3) *Shifting*: total revisits per AOI (i.e., number of fixations returning to the given AOI). The three attentional indices: gaze duration, first fixation location, and revisits were used as dependent variables.

### Statistical Analyses

We conducted separate linear mixed-effects analyses for each different attentional variable. Visual inspection of residual plots did not reveal any obvious deviations from homoscedasticity or normality for the between-subjects factor ‘Group’ (ASD, CD, RD, ASD+CD/RD, and HC) and the within-subjects factors stimulus categories ‘Social’ (non-social vs. social), and ‘Valence’ (positive vs. negative). These variables were included as fixed effects in each model. As a random effect, the intercepts for the individual participants were included. Gender, age and education level were included as covariates in all analyses, since these variables can affect mood disorders and attentional processing (Bjelland et al. 2008; Isaacowitz et al. 2006; Kendler et al. 2004). Data were analysed using Statistical Package of the Social Sciences (SPSS), version 25.0.

## Results

### Participants

The five groups did not differ significantly with respect to gender or age; however, the participants did differ significantly on education level (see Table 1). The depressed participants (CD and RD) had lower education levels compared to the ASD, ASD+CD/RD, and HC groups.

### Group Comparisons on Gaze Duration for Affective Social and Non-Social Information

#### Testing the Inclusion of the Random Intercept in the Model

To test the model fit, the likelihood ratio of the full model with the random effect was tested against the likelihood ratio of the model without the random effect, resulting in a significant difference, χ^2^ (1) = 634.78, *p* < 0.001. Thus, adding the random intercept significantly improved the fit of the model.

#### Linear Mixed Effects Analysis of Gaze Duration

A linear mixed effects analysis was conducted with the dependent variable Gaze Duration, the between-subjects factor Group and the within-subjects factors stimulus categories (Social and Valence) and the random intercept for Participant. The three-way interaction between Group, Social, and Valence was not significant, *F *(4, 13,102.08) = 1.88, *p* = 0.111. The interaction between Group and Valence was significant, *F *(4, 13,102.08) = 9.0, *p* < 0.001, as was the interaction between Valence and Social interaction, *F *(1, 13,102.08) = 16.69, *p* < 0.001. The interaction between Group and Social was not significant, *F *(4, 13,237.25) = 0.71, *p* = 0.586. The analysis further revealed a main effect of Valence, which indicated that participants looked longer at negative compared to positive stimuli, *F *(1, 13,102.08) = 9.70, *p* = 0.002, mean differences (*M*_diff_) = 190.64 ms, 95% CI [70.67, 310.61]. The main effects of Group and Social were not significant, Group: *F *(4, 145.97) = 1.96, *p* = 0.104; Social: *F *(1, 13,246.40) = 0.028, *p* = 0.867.

**Fig. 2:**
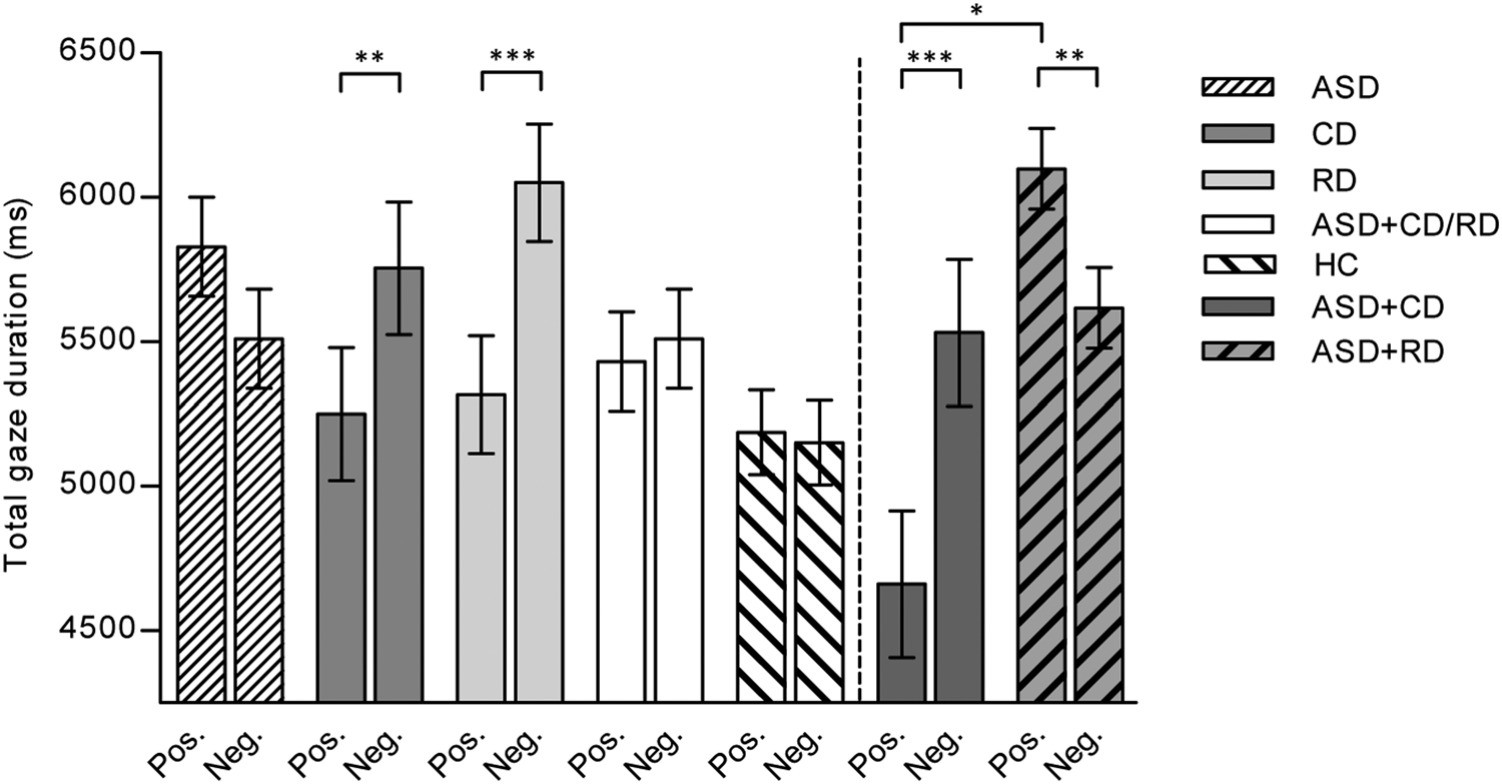
Total gaze duration (ms) per valence (positive vs. negative) for each group, *ASD* autism spectrum disorder, *CD* current depression, *RD* remitted depression, *ASD+CD/RD* autism spectrum disorder with depression, *HC* healthy controls, *ASD+CD* autism spectrum disorder with current depression, *ASD+RD* autism spectrum disorder with remitted depression. Asterisks highlight significantly different mean comparisons (*p < .05, **p < .01, ***p < .001). Error bars represent standard errors

Post hoc analyses of the Group*Valence interaction were conducted. First, we looked within each group to compare the total gaze duration for positive and negative pictures (collapsed over the social and non-social blocks). Both the CD and RD group had a significantly longer total gaze duration for negative than positive stimuli, RD: *F *(1, 2088.72) = 30.55, *p* < 0.001, *M*_diff_ = 732.82 ms, 95% CI [472.82, 992.82]; CD: *F *(1, 1613.87) = 9.9,* p* = 0.002, *M*_diff_ = 533.71 ms, 95% CI [200.90, 866.51]. The total gaze duration for positive and negative pictures did not differ significantly in the other groups (ASD, ASD+CD/RD and HC): ASD: *F *(1, 1281.15) = 2.46, *p* = 0.117, *M*_diff_ = − 318.70, 95% CI [−717.36, 79.95]; ASD+CD/RD: *F *(1, 2989.95) = 0.44, *p* = 0.510, *M*_diff_ = 75.85, 95% CI [−149.62, 301.32]; HC: *F *(1, 5127.31) = 0.13, *p* = 0.718, *M*_diff_ = − 29.22, 95% CI [−187.84, 129.40]. When comparing all groups on negative stimuli and positive stimuli only, we did not find group differences, *F *(4, 147.64) = 1.56, *p* = 0.189 and positive stimuli, *F *(4, 146.16) = 1.26, *p* = 0.287. Subsequently, post hoc analyses of the Valence*Social interaction revealed that, across groups, participants looked longer at negatively valenced social than non-social stimuli, *F *(1, 6569.66) = 10.95, *p* = 0.001, *M*_diff_ = − 243.40, 95% CI [−387.61, −99.19] and longer at positively valenced non-social than social stimuli, *F *(1, 6591.62) = 10.91, *p* = 0.001, *M*_dif_ = 245.19, 95% CI [99.69, 390.68]. No other post hoc comparisons reached significance, with all *p*-values  > 0.561. See Fig. 2 for the total gaze durations per valence for each group.

#### Depressive Symptoms Included as Covariate in the Linear Mixed Effects Analysis of Gaze Duration

Because the IDS-SR scores of the ASD group (mean: 25; clinical range 14 – 26, indicating mild depressive symptoms) are significantly higher compared to HC (mean: 5; clinical range 0 – 14, indicating no depressive symptoms), see also Table 1, we wanted to examine whether the negative bias found in ASD could be explained by comorbid (subclinical) depressive symptoms. Therefore, the IDS-SR total scores were added as a covariate in the linear mixed effects analysis of gaze duration. Entering the depressive symptoms as a covariate did not alter the within group differences found: both the CD and RD group looked significantly longer at negative than positive stimuli.

#### Examining the Correlations of Symptom Severity Scores of Depression and ASD with Total Gaze Duration

Visual attention patterns might vary with either depressive and/or ASD symptom severity scores. We, thus, examined the correlations between the symptom severity scores of ASD (AQ-50) and depression (IDS-SR) and attentional bias for social and non-social stimuli. Therefore, two bias scores were calculated (i.e., the total gaze duration for negative stimuli (in ms) minus the total gaze duration for positive stimuli (in ms) for both social and non-social categories). Neither the IDS-SR or AQ-50 total scores correlated significantly with these bias scores for social (IDS-SR: *r *(3454) = − 0.03, *p* = 0.081; AQ-50: r (3454) = − 0.03, *p* = 0.052) and non-social stimuli (IDS-SR: *r *(3310) =  − 0.01, *p* = 0.787; AQ-50: r (3310) =  − 0.02, *p* = 0.399).

#### Comparing ASD Participants with Current vs. Remitted Depression

We further divided the ASD+CD/RD group into ASD+CD and ASD+RD groups to examine whether the bias in ASD remains after remission (see Fig. 2 for the group means). The ASD+CD group (*n* = 15; 33% female) had a mean age of 39 years (*SD* = 16.29) and were 7% low, 60% middle, and 20% highly educated. The ASD+RD group (*n* = 22; 37% female) had a mean age of 34 years (*SD* = 10.73) and were 23% low, 27% middle, and 46% highly educated. The groups did not differ significantly on gender, age, and education level, gender: χ^2^(1) = 0.04, *p* > 0.850; age: *F *(1, 35) = 1.40, *p* > 0.246; education level: χ^2^(2) = 5.46, *p* = 0.065.

A linear mixed effects analysis was conducted with the dependent variable Gaze Duration, the between-subjects factor Group (ASD+CD and ASD+RD) and within-subjects factors stimulus categories (Social and Valence) and the random intercept for Participant.

This analysis revealed no significant three-way interaction between Group, Social, and Valence, *F *(1, 2990.01) = 0.72, *p* = 0.397. A significant interaction effect between Group and Valence was found, *F *(1, 2990.01) = 33.61, *p* < 0.001. The other interaction effects were not significant, Group*Social: *F *(1, 3022.98) = 0.10, *p* = 0.322; Valence*Social: *F *(1, 2990.01) = 2.01, *p* = 0.156. The main effects of Group, Valence, and Social were not significant, Group: *F *(1, 33.62) = 1.49, *p* = 0.231; Valence: *F *(1, 2990.01) = 2.90, *p* = 0.089; Social: *F *(1, 3022.98) = 0.34, *p* = 0.562.

Post hoc analyses revealed that participants with ASD+CD had longer total gaze durations towards negative than positive stimuli, *F *(1,1235) = 20.41, *p* < 0.001, *M*_diff_ = 870.37, 95% CI [492.38, 1248.35], while the ASD+RD participants showed significantly longer total gaze durations for positive stimuli than negative stimuli, *F *(1,1755.27) = 11.89, *p* = 0.001, *M*_diff_ = -482.46, 95% CI [−756.84, −208.08]. A significant difference between the groups for positive stimuli was found; the ASD+RD participants looked longer at positive stimuli than did the ASD+CD participants, *F*(1, 33.98) = 4.92, *p* = 0.033, *M*_diff_ = − 1089.64, 95% CI [− 2087.63, − 91.65]. No significant results were found for negative stimuli, *F *(1, 34.20) = 0.17, *p* = 0.682. For an overview of all means, standard errors and 95% CIs for the three-way interactions across all analyses per attentional index, see the Appendix Table 3.

### First Fixation Location

Because we aimed to compare the diagnostic groups, only analyses qualified by significant group interactions for both the first fixation location and revisits indices will be presented**.** In addition, due to the blocked design of the task, a comparison between the social and non-social stimuli does not make sense for the dependent variable First Fixation Location (i.e., all stimuli were social in the social block and non-social in the non-social block). Therefore, Social was not included as a factor in these analyses.

The random effect in the full model did not improve the fit of the model significantly, χ^2^(1) = 2.00, *p* > 0.05; thus, we did not include this random effect. The Group*Valence interaction was not significant, *F *(4, 574.00) = 1.84, *p* = 0.120, indicating that the groups oriented their initial attention to the valenced stimuli in a comparable manner.

#### IDS-SR as a Covariate in the Linear Mixed Effects Analysis of the Location of the First Fixation

The IDS-SR scores of the participants were included as an additional covariate in a post hoc linear mixed effects analysis of the location of the first fixation. Including the IDS-SR as an additional covariate did not alter the results significantly. The groups did not differ in allocating their initial attention to the valenced stimuli.

### Revisits

Including the random effect in the full model improved the fit of the model significantly, χ^2^(1) = 1949.61, *p* < 0.001. A linear mixed effects analysis showed no significant three-way interaction of Group, Valence, and Social, *F *(4, 12,661.02) = 0.75, *p* = 0.556. No interaction effects were found, Group*Valence: *F *(4, 12,661.10) = 1.55, *p* = 0.186; Group*Social: *F *(4, 12,768.60) = 0.13, *p* = 0.972.

#### IDS-SR as a Covariate in the Linear Mixed Effects Analysis of the Revisits

The IDS-SR scores of the participants were added as an additional covariate in a post hoc linear mixed effects analysis of the revisits variable, in which we entered IDS-SR as a covariate; this did not change the results significantly. The groups did not differ significantly in the three-way interaction of Group, Valence, and Social and in the two-way interaction effects (i.e., Group*Valence and Group*Social).

## Discussion

The purpose of this eye-tracking study was to gain insight in cognitive factors contributing to the high comorbidity of depression in ASD. We examined whether ASD is characterised by a negative attentional bias and whether this can be explained by comorbid clinical and subclinical depression. Three different component processes of attentional bias (overall engagement, initial engagement, and shifting) were investigated. With regard to the total gaze duration (overall engagement), we replicated previous research (e.g., Peckham et. al. 2010) demonstrating that both remitted and currently depressed participants show a negative attentional bias, possibly indicating difficulty in disengaging from negative stimuli (Gotlib and Joormann 2010). Considering a negative attentional bias is still present after remission in our neurotypical depressed sample, this bias might function as a trait-like characteristic in individuals vulnerable to depression (e.g., De Raedt and Koster 2010; Gotlib and Joormann 2010). A negative bias (i.e., longer gaze durations) was also found in the ASD participants with a current comorbid depression, corroborating previous findings in currently depressed individuals with comorbid psychopathologies (Dozois and Dobson 2001; LeMoult and Joormann 2012; Vrijsen et al. 2017). Such a depressotypic attentional bias was not observed in the whole group of participants with ASD or in healthy controls, as they showed no significant differentiation in attentional bias. This finding of the ASD participants is in contrast to the results of Unruh et al. (2018); however, this might be due to the inclusion of varying types of stimuli (i.e., valenced [non-]social scenes versus emotional or neutral facial expressions). Differences in the types of stimuli used in attentional paradigms may affect the observed attentional bias (Peckham et al. 2010). Finally, because examining the influence of subclinical depressive symptom levels on the gaze pattern is informative for the depression specificity of our results, especially with regard to the attentional bias pattern in ASD, we included the IDS-SR total score. This showed that the results of this study were independent of the presence of subclinical depression. Moreover, because patterns of visual attention may vary with either depressive and/or ASD symptoms independent of the diagnostic group, we examined the correlations between the symptom severity scores of ASD (AQ-50) and depression (IDS-SR) and attentional bias (as measured with the overall engagement index) for social and non-social stimuli. This showed that neither the IDS-SR or AQ-50 total scores correlated significantly with the bias scores for social and non-social stimuli. Thus, the relationship between negative attentional bias and the diagnostic groups does not extend beyond the depression or ASD diagnosis border. So, it might be that attentional bias is not a characteristic for the disorder-specific symptom severity indices as measured in this study.

Exploratory additional analyses demonstrated a negative bias in ASD participants with a comorbid current depression, but not in ASD participants remitted from depression. In fact, remitted depressed ASD participants looked longer at positive than negative stimuli. The results suggests that a similar gaze duration pattern was found in never-depressed ASD participants. Although these participants did not differ significantly from controls, it is noteworthy that the unstandardised effect size of this group comparison was comparable to that of the significant difference between CD and RD on attentional bias. These findings may imply that a depressotypic attentional bias might not persist beyond a depressive episode in individuals with ASD. ASD individuals might be characterised by a rather positive information processing style. Therefore, this possible premorbid positive information processing style of the ASD individuals might be less affected by the lingering effect a depressive episode can have on bias, as seen in the neurotypical remitted depressed individuals (i.e., indicated by the presence of a persistent negative attentional bias).

Further, the healthy controls did not show a differential attentional bias towards valenced stimuli for any of the attentional indices, in contrast to our expectations and previous findings (e.g., De Raedt and Koster 2010). An explanation for the lack of a positive bias in the healthy controls might be that the negative stimuli were more arousing in this study, and therefore attracted relatively more attention (Vogt et al. 2008). In addition, the groups showed no differences between social/non-social valenced content of the stimuli for any of the attentional indices, which is likely due to the blocked design of the task (i.e., presenting the social and non-social blocks separately, thus non-competing).

The groups did not differ on the initial or shifting indices. All individuals allocated their initial fixation on and number of revisits towards the different affective stimuli in a similar fashion, which is in line with previous research (Armstrong and Olatunji 2012; Gotlib and Joormann 2010). In contrast, Santos et al. (2012) demonstrated differences between the location of the first fixation in individuals with ASD and controls; the ASD individuals did not have an initial preference for the negative threatening social stimuli compared to TD. However, the negative stimuli included in their study were threating social scenes, which were compared to neutral social scenes, instead of the more general negative scenes (i.e., threatening, dysphoria-related, and disgusting scenes) that were compared to positive scenes in our study. This could explain the differences between our results and those by Santos et al. (2012). Moreover, the location of the first fixation is a vigilance based outcome measure, which is generally associated with anxiety disorders (Gamble and Rapee 2010; Mogg and Bradley 2005).

If replicated, the present findings may have implications for clinical practice. Current treatment innovations designed for depressed individuals that target negative bias by means of computerised (add-on) treatment, such as Attentional Bias Modification (ABM), can be administered in addition to treatment as usual. Since we found a negative bias in currently depressed individuals with ASD, ABM could likewise be beneficial for these individuals. Offering a tailored (add-on) ABM treatment based on a patient’s diagnosis may expand the limited efficacious treatment options specifically designed for this patient group. Subsequently, ABM, or a similar treatment option, may also be used to index (symptoms of) current depression in ASD, and may thereby possibly aid in improving the problem of underdiagnosis of depression in ASD. This needs more research. Since our study demonstrated that, in addition to the well-investigated neurotypical depressed individuals, the presence of negative attentional bias may extend to currently depressed ASD participants, future studies could advance our knowledge about underlying mechanisms, such as attentional bias, of comorbid mental disorders, by investigating the association between attentional bias and severity levels of symptoms of mental disorders. This is in line with initiatives such as the National Institute of Mental Health’s Research Domain Criteria project (RDoC; NIHM 2008) and is aiming towards a transdiagnostic approach to mental health.

A strength of the current study is the inclusion of a well-defined naturalistic clinical patient sample which facilitates generalization of our findings to the clinical population in which comorbidity is common (Kessler et al. 2005). A limitation is the limited number of never-depressed participants with ASD in this study, thus, caution regarding the interpretation of this result is advised and the study is in need of replication. Because, the unstandardised effect size of the group comparison of never-depressed ASD and remitted depressed ASD was comparable to the significant difference found in attentional bias between CD and RD. The current study is a first step to help explain the high comorbidity of depression in ASD, and will hopefully instigate further research into attentional bias and other cognitive markers in ASD. Moreover, the neurotypical depressed individuals were lower educated than the individuals with ASD (and depression) and healthy controls in the current study, which may have influenced the results. To further explore attentional bias for social stimuli, a future study could include competing social and non-social, positive and negative stimuli within each slide. For this first exploration, we chose to present the social and non-social stimuli in a blocked design. In conclusion, attentional bias to negatively valenced stimuli seems to be only present in ASD individuals with current depression and is most likely absent in ASD who are currently not depressed (either in remission or never-depressed). This suggests that negative attentional bias might be a depression state-specific marker only for currently depressed ASD individuals.

## Appendix

See Table 3.

**Table 3:** Means, standard errors (SE) and 95% Confidence intervals (CI) for the three-way interactions between Group, Valence and Social for the three attentional indices (gaze duration (ms), first fixation location and revisits)

Group	Valence		(Non- social)		*M*			SE		95% CI
*Gaze duration (ms)*										
HC	Negative		Non-social		5034.27			159.59		[4719.93, 5348.61]
			Social		5268.82			158.27		[4957.03, 5580.60]
	Positive		Non-social		5373.06			159.59		[5058.72, 5687.40]
			Social		4999.72			158.27		[4687.93, 5311.51]
CD	Negative		Non-social		5558.13			258.93		[5048.57, 6067.69]
			Social		5950.05			250.99		[5455.87, 6444.22]
	Positive		Non-social		5555.23			258.93		[5045.67, 6064.79]
			Social		4944.51			250.99		[4450.34, 5438.69]
RD	Negative		Non-social		5977.39			228.66		[5527.37, 6427.41]
			Social		6124.28			223.36		[5684.53, 6564.02]
	Positive		Non-social		5213.02			228.66		[4763.00, 5663.04]
			Social		5420.27			223.36		[4980.52, 5860.02]
ASD	Negative		Non-social		5448.55			292.19		[4873.40, 6023.71]
			Social		5736.65			286.89		[5171.77, 6301.53]
	Positive		Non-social		6062.49			292.19		[5487.33, 6637.64]
			Social		5781.21			286.89		[5216.33, 6346.09]
ASD+CD/RD	Negative		Non-social		5389.03			189.88		[5015.24, 5762.83]
			Social		5630.41			191.09		[5254.25, 6006.57]
	Positive		Non-social		5500.67			189.88		[5126.87, 5874.46]
			Social		5361.03			191.09		[4984.87, 5737.18]
*Post hoc analysis*										
ASD+CD	Negative		Non-social		5533.73			324.34		[4883.81, 6183.65]
			Social		5786.67			324.34		[5136.76, 6436.59]
	Positive		Non-social		4729.51			324.34		[4079.59, 5379.43]
			Social		4850.16			324.34		[4200.24, 5500.07]
ASD+RD	Negative		Non-social		5311.51			255.25		[4801.36, 5821.67]
			Social		5525.62			258.01		[5010.29, 6040.95]
	Positive		Non-social		6049.78			255.25		[5539.63, 6559.94]
			Social		5738.06			258.01		[5222.73, 6252.38]
*First fixation location*										
HC	Negative		Non-social		7.10			0.22		[6.67, 7.54]
			Social		5.93			0.22		[5.50, 6.36]
	Positive		Non-social		4.88			0.22		[4.45, 5.32]
			Social		6.08			0.22		[5.65, 6.51]
CD	Negative		Non-social		6.37			0.26		[5.86, 6.88]
			Social		6.33			0.26		[5.83, 6.84]
	Positive		Non-social		5.63			0.26		[5.12, 6.14]
		Social		5.67			0.26		[6.20, 7.52]	
RD	Negative	Non-social		6.86			0.34		[6.29, 7.42]	
		Social		5.48			0.32		[4.85, 6.11]	
	Positive	Non-social		5.15			0.34		[4.49, 5.80]	
		Social		6.52			0.32		[5.90, 7.15]	
ASD	Negative	Non-social		7.67			0.89		[5.93, 9,41]	
		Social		4.00			0.89		[2.20, 5.74]	
	Positive	Non-social		4.32			0.89		[2.59, 6.07]	
		Social		8.00			0.89		[6.26, 9.74]	
ASD+CD/RD	Negative	Non-social		6.89			0.27		[6.34, 7.41]	
		Social		6.33			0.28		[5.78, 6.90]	
	Positive	Non-social		5.08			0.27		[4.53, 5.60]	
		Social		5.67			0.28		[5.11, 6.22]	
*Post hoc analysis*										
ASD+CD	Negative	Non-social		7.62			0.43		[6.76, 8.48]	
		Social		6.54			0.43		[5.69, 7.40]	
	Positive	Non-social		4.39			0.43		[3.53, 5.24]	
		Social		5.46			0.43		[4.61, 6.32]	
ASD+RD	Negative	Non-social		6.41			0.36		[5.67, 7.08]	
		Social		6.18			0.37		[5.45, 6,92]	
	Positive	Non-social		5.56			0.36		[4.83, 6.24]	
		Social		5.82			0.37		[5.10, 6.57]	
*Revisits*										
HC	Negative	Non-social		2.00			0.11		[1.78, 2.22]	
		Social		2.03			0.11		[1.81, 2.25]	
	Positive	Non-social		2.19			0.11		[1.97, 2.42]	
		Social		2.08			0.11		[1.86, 2.30]	
CD	Negative	Non-social		1.77			0.18		[1.42, 2.12]	
		Social		1.77			0.18		[1.43, 2.12]	
	Positive	Non-social		1.90			0.18		[1.54, 2.25]	
		Social		1.72			0.18		[1.37, 2.07]	
RD	Negative	Non-social		2.06			0.16		[1.75, 2.38]	
		Social		2.00			0.16		[1.69, 2.31]	
	Positive	Non-social		2.18			0.16		[1.86, 2.49]	
		Social		2.08			0.16		[1.77, 2.39]	
ASD	Negative	Non-social		2.02			0.20		[1.62, 2.42]	
		Social		1.97			0.20		[1.57, 2.36]	
	Positive	Non-social		2.30			0.20		[1.90, 2.70]	
		Social		2.28			0.20		[1.88, 2.68]	
ASD+CD/RD	Negative	Non-social		1.96		0.13			[1.70, 2.22]	
		Social		2.05		0.13			[1.79, 2.31]	
	Positive	Non-social		2.20		0.13			[1.93, 2.46]	
		Social		2.03		0.13			[1.76, 2.29]	
*Post hoc analysis*										
ASD+CD	Negative	Non-social		2.13		0.24			[1.63, 2.62]	
		Social		2.20		0.24			[1.70, 2.69]	
	Positive	Non-social		2.13		0.24			[1.64, 2.63]	
		Social		2.14		0.24			[1.64, 2.63]	
ASD+RD	Negative	Non-social		1.94		0.19			[1.56, 2.32]	
		Social		2.04		0.19			[1.66, 2.42]	
	Positive	Non-social		2.34		0.19			[1.96, 2.72]	
		Social		2.04		0.19			[1.66, 2.43]	
